# Tetrel Bonding Interactions in Perchlorinated Cyclopenta- and Cyclohexatetrelanes: A Combined DFT and CSD Study

**DOI:** 10.3390/molecules23071770

**Published:** 2018-07-19

**Authors:** Antonio Bauzá, Antonio Frontera

**Affiliations:** Department of Chemistry, Universitat de les Illes Balears, Crta de Valldemossa km 7.5, 07122 Palma de Mallorca (Baleares), Spain

**Keywords:** tetrel bonding interactions, CSD search, DFT calculations, AIM analysis

## Abstract

In this manuscript, we combined DFT calculations (PBE0-D3/def2-TZVP level of theory) and a Cambridge Structural Database (CSD) survey to evaluate the ability of perchlorinated cyclopenta- and cyclohexatetrelanes in establishing tetrel bonding interactions. For this purpose, we used Tr_5_Cl_10_ and Tr_6_Cl_12_ (Tr = Si and Ge) and HCN, HF, OH^−^ and Cl^−^ as electron donor entities. Furthermore, we performed an Atoms in Molecules (AIM) analysis to further describe and characterize the interactions studied herein. A survey of crystal structures in the CSD reveals that close contacts between Si and lone-pair-possessing atoms are quite common and oriented along the extension of the covalent bond formed by the silicon with the halogen atom.

## 1. Introduction

The fascinating progress achieved in modern chemistry during the last decade has been supported by an in-depth understanding of noncovalent interactions, which are the pillars of supramolecular chemistry [1,2]. Therefore, their proper comprehension is key for chemists working in this area of research, since many chemical and biological processes are regulated by a precise combination of noncovalent forces, which often dictate the pathway of highly specific recognition mechanisms. For instance, the formation process of novel supramolecular assemblies is usually governed by an intricate combination of interactions between hosts and guests, presenting high affinities, even in highly competitive media [3,4,5,6]. For this reason, it is necessary to adequately describe and understand noncovalent interactions between molecules to achieve progress in this field of research. In this context, hydrogen bonding interactions are known as a classical supramolecular force present in many chemical and biological environments [7]. Similarly, halogen bonding interactions [8] have been found to share both strength and directionality features with hydrogen bonds. Consequently, the Cambridge Structural Database (CSD) was inspected in a series of studies in order to gain some insights into the impact of this interaction in solid state chemistry [9,10]. The scientific interest regarding this interaction has expanded exponentially due to its recognition as a prominent player in biological media and the design of new materials; leading to a wide amount of theoretical and experimental studies [11,12,13,14]. In addition, it has been widely recognized that σ-holes (and more recently π-holes [15]) can also appear in positive electrostatic potential regions involving covalently bond atoms of groups III to VIII [16,17,18,19,20,21,22]. Besides, several theoretical studies have focused on the study of their physical nature [23,24,25,26,27], concluding that it is basically sustained by the interaction of an electron-rich entity (electron donor) with a σ-hole (electron acceptor), in a close way to hydrogen and halogen-bonding interactions [7,12].

In this regard, the recognition of tetrel-bonding interactions [28] (i.e., an attractive noncovalent force between a σ-/π-hole present in a group IV atom and a Lewis base) has increased among the scientific community over the past years. In particular, both experimental [24,29] and theoretical [30,31] chemists have contributed to expanding current knowledge by evaluating their impact on solid state, ref. [32] biological systems [33] and chemical reactivity [34]. Of particular interest among the scientific community is perhalogenated cyclohexasilanes, due to its ability to act as a multiple tetrel bond donor using the twelve available σ-holes. In fact, crystallographic studies [35,36,37] have shown that perhalogenated cyclohexasilanes can strongly bind electron-rich moieties, such as halide anions or organocyanides (such as acetonitrile). Several theoretical studies [38,39,40,41] have explored this possibility by theoretically analyzing a series of anion/lone pair-Si inverted sandwiched complexes and confirming their ability to behave as efficient ditopic anion receptors.

**Figure 1:**
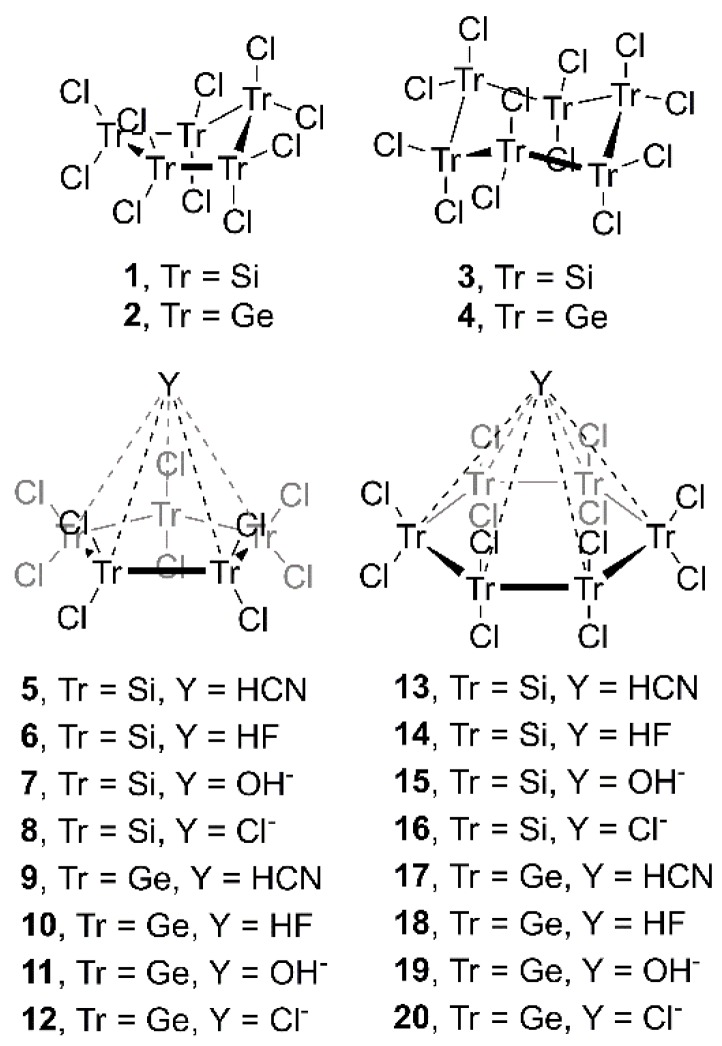
Compounds and complexes **1**–**20** studied in this work.

In this context, we wondered about the possibility of (i) expanding current knowledge to cyclopentatetrelanes (Si and Ge) and (ii) exploring the effect of Ge in cyclohexa-derivatives. In order to achieve this goal, we used Tr_5_Cl_10_ and Tr_6_Cl_12_ molecules, where Tr = Si and Ge, and HCN, HF, OH^−^ and Cl^−^ moieties, as neutral and anionic electron donors, respectively (see Figure 1). In addition, we performed an Atoms in Molecules (AIM) analysis to further characterize the interactions described herein. Finally, we carried out a CSD survey in order to find experimental evidence of the importance of tetrel bonding interactions in the solid state involving perhalogenated cyclopenta- and cyclohexatetrelanes.

## 2. Results and Discussion

### 2.1. Preliminary MEP Analysis

**Figure 2:**
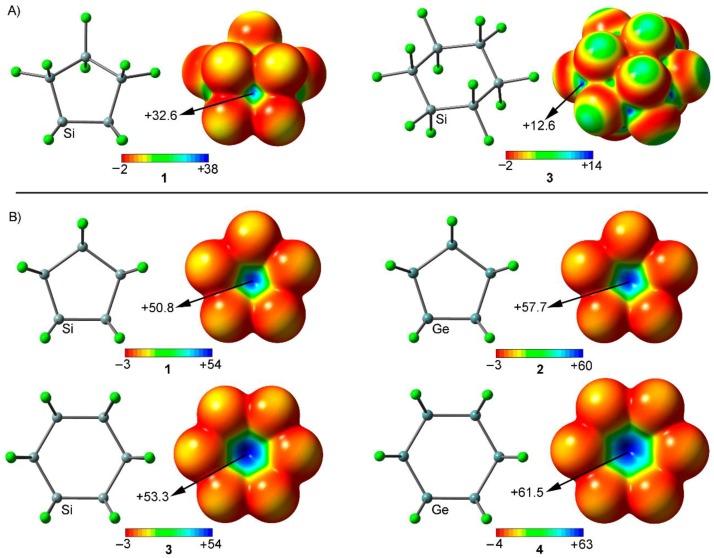
(**A**) MEP surfaces of compounds **1** and **3** in envelope and chair conformations, respectively. (**B**) MEP surfaces of compounds **1** to **4** in a planar disposition. Energies at selected points of the surface (0.001 a.u.) are given in kcal/mol.

We firstly computed the molecular electrostatic potential (MEP) mapped onto the van der Waals surface for compounds **1** and **3** in their respective envelope and chair conformations (Figure 2A). As noted, both molecules show areas of positive electrostatic potential on extension of the Si-Cl and Si-Si bonds, named σ-holes. Particularly, in case of **1**, the most positive MEP region is located at one face of the molecule (the face opposite to the axial Cl atom bonded to the *endo* carbon atom). This region of positive MEP is formed by the superposition of four Si-Cl σ-holes (see Figure 2A, left). On the other hand, in case of compound **3**, six small σ-holes at the extension of the six Si–Cl axial bonds can be observed. The MEP value at these symmetrically distributed σ-holes is significantly smaller (12.6 kcal/mol) that that at the s-hole of the five membered ring, because only one Si–Cl bond is involved. In addition, both molecules present a low σ-hole accessibility, since they are closely surrounded by four (in **1**) and three (in **3**) negative belts belonging to the chlorine substituents, which disfavor the interaction with electron rich species, owing to both electrostatic and steric repulsive effects. However, when a planar disposition is imposed (see Figure 2B), the σ-holes gain in both magnitude size as well as become more accessible, thus enhancing the interaction with electron rich guests from both electrostatic and steric perspectives.

As noted in Figure 2B, in all cases, a positive electrostatic potential region can be located on the center of the ring, as a consequence of the combination of five (in **1** and **2**) and six (in **3** and **4**) Cl-Tr σ-holes (Tr = Si, Ge). The presence of this region ensures an attractive interaction with an electron-rich entity. In addition, the MEP values at the center of the ring are more positive for Ge derivatives (compounds **2** and **4**) than for their Si analogous (compounds **1** and **3**), thus expecting more favorable interaction energy values for complexes involving the former, as it is known for other σ-hole interactions [16]. It is also worthy to note than the MEP values are more positive for six membered rings (compounds **3** and **4**), due to the participation of an additional Cl-Si σ-hole, thus anticipating larger interaction energy values from an electrostatic point of view.

### 2.2. Energetic and Geometric Results

**Figure 3:**
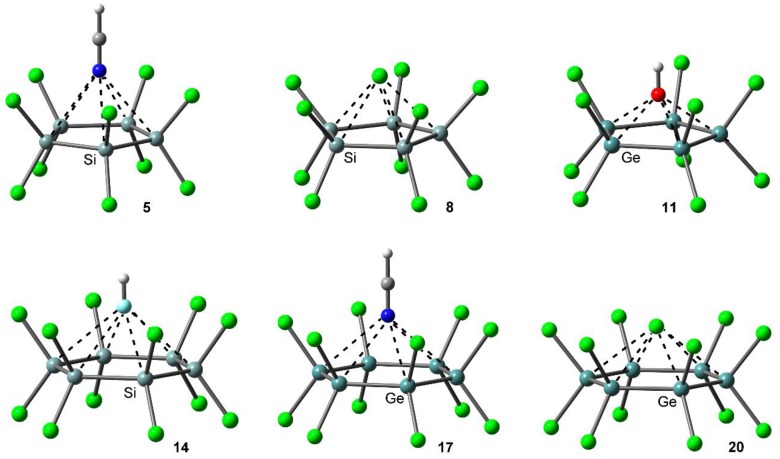
PBE0-D3/def2-TZVP optimized geometries of complexes **5**, **8**, **11**, **14**, **17** and **20**.

Table 1 gathers the interaction energies and equilibrium distances of optimized complexes **5** to **20** (see Figure 3), computed at the PBE0-D3/def2-TZVP level of theory. From analysis of the results, several points arise. First, in all cases with the exception of complexes **13** and **14**, the interaction energy values are favorable and vary from moderately strong (in case of neutral donors) to strong (in case of charged donors), ranging between −107.5 and −3.7 kcal/mol. Second, complex **11** involving OH^−^ obtained the most favorable interaction energy value of the study, while complex **14** involving HF obtained the poorest binding energy value of the study. Finally, complexes involving Ge (**9** to **12** and **17** to **20**) achieved larger interaction energy values than those involving Si (**5** to **8** and **13** to **16**), in agreement with the MEP analysis discussed above.

For complexes involving perchlorinated cyclopentatetrelanes (**5** to **12**), complexes **9** and **11** involving HCN and OH^−^ obtained the largest interaction energy values of their respective series (−11.4 and −107.5 kcal/mol). On the other hand, complexes **6** and **10** achieved the poorest binding energy values of the series, owing to the low basicity of the HF molecule (−3.7 and −7.7 kcal/mol, respectively). Finally, complexes **8** and **12** involving Cl^−^ obtained a lower interaction energy value than their OH^−^ analogous (−61.4 and −68 kcal/mol, respectively), due to the higher basicity of the latter.

Among complexes **13** to **20** involving perchlorinated cyclohexatetrelanes, a similar behavior is observed in case of charged complexes **15**, **16**, **19** and **20**, where those involving OH^−^ (**15** and **19**) obtained a larger interaction energy value (−95.3 and −106.3 kcal/mol) than those involving Cl^−^ (complex **16**, −59.3 kcal/mol and complex **20**, −70 kcal/mol). On the other hand, in case of neutral complexes (**13**, **14**, **17** and **18**), those involving HCN as electron donor (**13** and **17**) obtained a more favorable binding energy value (−0.4 and −8.3 kcal/mol, respectively) than those involving HF (**14** and **18**), in agreement to that observed for complexes involving cyclopentatetrelanes. It is also worth noting that the magnitude of the interaction energy is almost negligible in case of complex **13** and repulsive in case of complex **14** (+3.2 kcal/mol). For these complexes, we computed the 1:2 assemblies (one cyclohexasilane and two lone pair donor molecules, denoted as **13A** for HCN and **14A** for HF), obtaining favorable interaction energy values of −12.6 kcal/mol for complex **13A** and −5.7 kcal/mol in case of complex **14A** (see Table 1). To further clarify the large difference between 1:1 and 1:2 complexes, we computed the energetic difference between the planar and the chair conformation in compound **3**, which is 12.3 kcal/mol. Therefore, the interaction of compound **3** with HCN (six concurrent tetrel bonds) is just able to compensate the difference between the chair and planar conformation, thus resulting in a negligible binding energy. The binding energy of the 1:2 complex (**13A**) is −12.2 kcal/mol, because six additional tetrel bonds are established (twelve in total, six in each side of the ring). In case of complex **14**, due to the lower basicity of the HF, the formation of the six Si···F tetrel bonds (1:1 complex) is not able to compensate the 12.3 kcal/mol required for changing the chair conformation into a planar one. Consequently, the 1:1 complex results to be 3.2 kcal/mol higher in energy than the separated monomers (only compensates around 9.1 kcal/mol). In good agreement, when the 1:2 complex (**14A**) is formed, the interaction energy becomes favorable (–5.7 kcal/mol) thus the additional six tetrel bonding interactions account for −8.9 kcal/mol. For the complexes of compound **4** (Ge instead of Si) all computed interaction energies are favorable because the difference in energy between the chair and planar conformation is only 6.8 kcal/mol.

Finally, it is also somewhat unexpected that complexes involving cyclopentatetrelanes (**5** to **12**) obtained more favorable binding energy values than their corresponding cyclohexatetrelane analogous (**13** to **20**), contrary to that obtained in the MEP analysis shown above for the planar molecules. Among other factors like proximity of the σ-holes and/or the negative belts of the chlorine atoms, the most likely explanation is that the difference in energy between the envelope and planar conformation is of 2.7 kcal/mol in **1** and 1.2 kcal/mol in **2**.

Although the interaction described above resembles lone pair–π (or anion–π) interactions) [42], where a positive electrostatic potential region located at the center of the aromatic moiety interacts with an electron rich moiety, we (and other research groups [41]) consider this particular interaction as a σ-hole bonding. That is, the positive electrostatic potential area emerges over the center of the ring as the superposition of six/five σ-holes at the extension of the Si/Ge–Cl covalent bonds.

### 2.3. AIM and NCI Analyses

**Figure 4:**
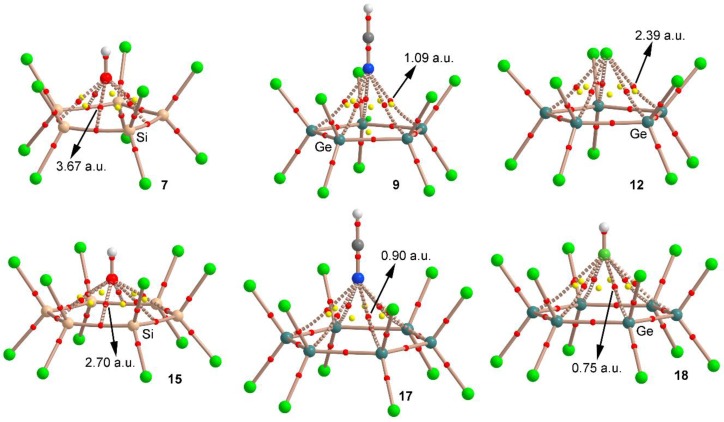
Distribution of critical points (red spheres) and bond paths for complexes **7**, **9**, **12**, **15, 17** and **18** at the PBE0/def2-TZVP level of theory. Bond, ring and cage CPs are represented by red, yellow and green spheres, respectively. The values of the charge density (ρ) at the bond critical points that emerge upon complexation are indicated in a.u.

We have used the Bader’s theory of “atoms in molecules” [43] (AIM) to characterize the noncovalent interactions shown in complexes **5**–**20**. A bond critical point (CP) and a bond path connecting two atoms is an unambiguous evidence of interaction. The AIM distribution of critical points and bond paths computed for some representative examples are shown in Figure 4. As noted, for complexes involving cyclopentatetrelanes (**7**, **9** and **12**) five symmetrically distributed bond CPs interconnect the electron donor and tetrel atoms, thus characterizing five simultaneous tetrel bonding interactions.

On the other hand, in case of complexes **15**, **17** and **18** involving cyclohexatetrelanes, six symmetrically distributed bond CPs interconnect the electron donor atom and the tetrel atoms, which characterize six simultaneous tetrel bonding interactions. Furthermore, in all cases, several ring CPs emerge (five for complexes **7**, **9** and **12** and six for complexes **15**, **17** and **18**), due to the formation of several supramolecular rings, which further describe the interaction. It is also worthy to mention that in case of complex **9,** a cage CP is observed, which also describes the interaction. Curiously, in case of the Si compounds, the bond path connects the Si–Si bond CP to the electron rich atom. Finally, the value of the laplacian in all cases is positive, as is common in closed shell calculations.

**Figure 5:**
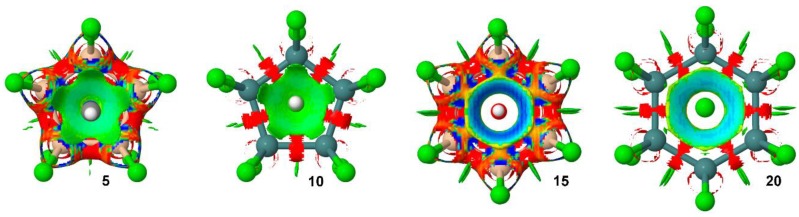
NCI plots of complexes **5**, **10**, **15** and **20**. The gradient cut-off is s = 0.35 au, and the color scale is −0.04 < ρ < 0.04 au.

We have also carried out an Non Covalent Interactions (NCI) plot [44] of some representative examples to further analyze the tetrel bonding complexes discussed above (see Figure 5). The NCI visualization index enables the identification and characterization of non-covalent interactions in an efficient way. The NCI plot allows an assessment of host–guest assembly complementarity and the extent to which weak interactions stabilize a complex. The information provided is basically qualitative, that is, which molecular regions are involved in the interaction. 

As noted, in case of complexes involving neutral donors (**5** and **10**), a green isosurface covers the entire cyclopentatetrelane moiety and characterizes the five simultaneous tetrel bonds. On the other hand, in case of anionic complexes **15** and **20,** the color of the isosurface is blue due to the existence of a strong electrostatic contribution to the interaction. Particularly, in case of complex **15,** the isosurface shows a more-pronounced blue region, in agreement with the strong interaction energy of complex **15** (see Table 1). In both complexes the isosurface is extended among all six σ-holes from the Si and Ge atoms. The nonexistence of surface at the center of the ring is in good agreement with the proposed σ-hole nature of the interaction instead of anion–π.

### 2.4. CSD Search

**Figure 6:**
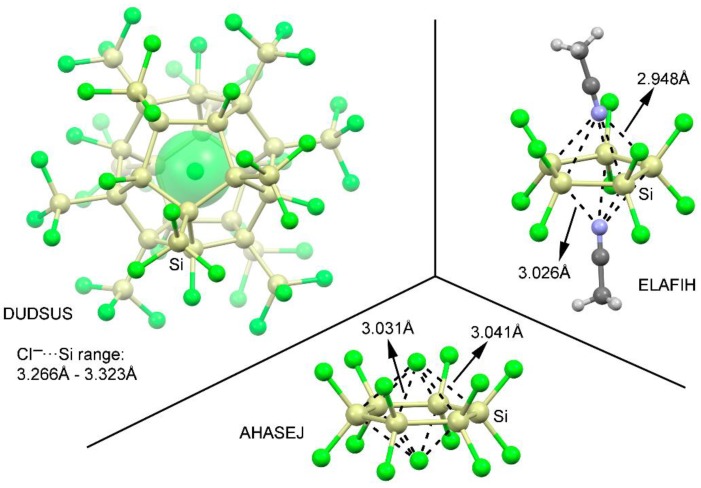
Partial views of the X-ray structure of some perchlorinated cyclopenta- and cyclohexasilanes establishing tetrel bonding interactions. The CSD codes are indicated.

We have explored CSD [45] to find evidence of the importance of tetrel bonding interactions involving perhalogenated cyclopenta- and cyclohexatetrelanes. During the search, we considered any sp^3^ tetrel atom apart from C (from Si to Pb) and any type of substituent in five and six membered rings. We found 11 structures containing cyclopentasilanes and 23 structures containing cyclohexasilanes (see Supplementary Materials—ESI for the complete list of structures). No structures were found involving other tetrel atoms. In addition, among these structures, 4 belonging to cyclopentasilane moieties and 19 structures involving cyclohexasilanes exhibit tetrel bonding interactions. Some examples are shown in Figure 6. In detail, in DUDSUS [46], the crystal packing consists of discrete [Si_32_Cl_45_]^–^ cluster units formed by a Si_20_ dodecahedral core bearing an endohedral Cl^–^ ion. Moreover, each Si_20_ core carries eight chloro and twelve trichlorosilyl substituents that fulfill all silicon cluster atom valencies. In addition, these electron withdrawing groups ensure the presence of Si σ-holes pointing inside of the cavity, leading to the establishment of multiple tetrel bonding interactions that act as a stabilizing source of the Cl^–^ ions. On the other hand, in ELAFIH [47] and AHASEJ [48] structures, the solid state architecture is governed by the formation of 2:1 dimers involving a perchlorinated cyclopenta- and cyclohexasilane rings and two acetonitrile and chloride molecules, respectively, in a 2:1 inverted sandwich fashion. It is also worthy to remark that experimentally only the 2:1 complexes are observed, in line with the energetic results obtained for complexes **13** and **14**. Finally, the distance values obtained are also within the range of the ones retrieved from the solid state, giving reliability to the theoretical results and highlighting the importance of these interactions in the solid state architecture of cyclopenta- and cyclohexasilanes.

## 3. Theoretical Methods 

The geometries of the complexes studied herein have been fully optimized at the PBE0-D3/def2-TZVP level of theory. The calculations were performed by using the program TURBOMOLE version 7.0 (University of Karlsruhe, Karlsruhe, Germany) [49]. The calculation of the interaction energy values was performed using the formula E_int_ = E_AB_ − E_A_ − E_B,_ where E_AB_ corresponds to the energy of the optimized complex, while E_A_ and E_B_ refer to the energies of the optimized isolated monomers. The *C*_5v_ or C_6v_ symmetry point groups were used in the optimization of the anionic complexes and no symmetry constrains were imposed in the neutral complexes. It should be mentioned that the geometries of the neutral complexes (stationary points) converge to *C*_s_ and *C*_2v_ point groups for the five-membered and six-membered rings, respectively. For some anionic complexes, we carried out optimization without imposing symmetry constraints and the final optimized geometries (stationary points) adopted either *C*_5v_ or C_6v_ symmetry. The interaction energies were calculated with correction for the basis set superposition error (BSSE) by using the Boys–Bernardi counterpoise [50]. The Bader’s “Atoms in molecules” theory was used to study the interactions discussed herein by means of the AIMAll calculation package (version 17.11.14, TK Gristmill Software, Overland Park, KS, USA) [51]. The calculations for the wavefunction analysis were performed by means of the Gaussian 09 calculation package (version B.01, Gaussian inc., Wallingford, CT, USA) [52]. The NCI plot is a visualization index based on electron density and its derivatives, and enables identification and visualization of non-covalent interactions. The isosurfaces correspond to both favorable and unfavorable interactions, as differentiated by the sign of the second density Hessian eigenvalue and defined by the isosurface color. The color scheme is a red-yellow-green-blue scale with red for ρ^+^_cut_ (repulsive) and blue for ρ^−^_cut_ (attractive). Yellow and green surfaces correspond to weak repulsive and weak attractive interactions, respectively. The models were designed based on previous theoretical studies that analyzed the ability of cyclohexasilanes to properly accommodate two anionic donor entities (mostly halogen ions, see [41]). We also included an OH^−^ ion and two neutral electron donors (HCN and HF molecules) to obtain a more representative set of complexes. In addition, we chose cyclohexagermanane and the cyclopenta- derivatives of Si and Ge as tetrel bond donors to gain further insights into the behavior of this family of compounds.

## 4. Conclusions 

In this manuscript, we analyzed the ability of perchlorinated cyclopenta- and cyclohexatetrelanes to establish tetrel bonding interactions with both neutral and charged electron donors. We used Tr_5_Cl_10_ and Tr_6_Cl_12_ (Tr = Si and Ge) and HCN, HF, OH^‒^ and Cl^‒^ moieties as electron donor molecules. In relation to this, complex **11** involving cyclopentagermanane and OH^‒^ as a Lewis base obtained the largest binding energy value of the study. On the other hand, complex **14** involving HF and cyclohexasililane achieved the poorest interaction energy value of the study. We also demonstrated that the energetic cost of forming the 2:1 complex compensates the energy penalty of passing from chair to planar conformation in cyclohexasilane complexes **13** and **14**. In addition, we have observed a reinforcement of the interaction strength ongoing from Si to Ge in both cyclopenta- and cyclohexatetrelane systems, as it is commonly observed for other σ-hole interactions. Furthermore, we performed Atoms in Molecules (AIM) analysis to further characterize the interactions described above. Finally, several experimental examples retrieved from the Cambridge Structural Database (CSD) were shown in order to provide reliability to the results and to highlight the importance of these interactions in the solid state of cyclopenta- and cyclohexatetrelanes.

## Figures and Tables

**Table 1:** Interaction energies without and with BSSE correction (ΔE and ΔE_BSSE,_ respectively, kcal/mol), equilibrium distances (R, Å) and value of the density at the bond CP (10^2^ × ρ, a.u.) for complexes **5**–**20** at the PBE0-D3/def2-TZVP level of theory.

Complex	ΔE ^a^	ΔE_BSSE_	R ^b^	10^2^ × ρ
**5**	−7.8	−7.1	2.350	1.17
**6**	−5.0	−3.7	2.271	0.94
**7**	−116.3	−102.4	1.401	3.67
**8**	−67.3	−61.4	2.067	2.72
**9**	−12.3	−11.4	2.340	0.97
**10**	−9.1	−7.6	2.237	0.81
**11**	−121.1	−107.5	1.430	3.36
**12**	−73.8	−68.0	2.121	2.39
**13**	−1.2	−0.5	2.145	1.09
**13A** ^a^	−14.1	−12.6	2.157	-
**14**	+1.9	+3.2	2.051	0.91
**14A** ^a^	−8.3	−5.7	2.062	-
**15**	−109.1	−95.3	1.223	2.70
**16**	−65.4	−59.3	1.849	2.23
**17**	−9.2	−8.3	2.118	0.90
**18**	−5.6	−4.1	2.021	0.75
**19**	−120.0	−106.3	1.196	2.46
**20**	−76.2	−70.0	1.870	1.97

^a^**13A** and **14A** are 1:2 complexes where two HCN and HF molecules are located above and below the Si_n_ molecular plane. ^b^ Distances measured from the electron rich atom to the ring centroid.

## Supplementary Materials

Supplementary Materials are available online, cartesian coordinates of the complexes and results from the CSD search.

## Abbreviations

The following abbreviations are used in this manuscript:

AIM	Atoms in molecules
MEP	Molecular electrostatic potential
BSSE	Basis Set Superposition Error
CSD	Cambridge Structural Database
CP	Critical point
NCIplot	Non Covalent Interactions plot
MINECO	Ministerio de Economía y Competitividad
AEI	Agencia Española de Investigación
