# Supplementary material for: Tetrel Bonding Interactions in Perchlorinated Cyclopenta- and Cyclohexatetrelanes: A Combined DFT and CSD Study
Source: Molecules. 2018 Jul 19;23(7):1770. doi: 10.3390/molecules23071770 (PMC6100242; doi:10.3390/molecules23071770)
Supplement: Supplementary file 1 [file molecules-23-01770-s001.pdf]

Tetrel bonding interactions in perchlorinated cyclopenta- and cyclohexatetrelanes: a combined DFT and CSD study.

Antonio Bauzá,[1*] and Antonio Frontera,[1]*

[1]Department of Chemistry Universitat de les Illes Balears, Crta. de Valldemossa km 7.5, 07122 Palma (Baleares), Spain. E-mail: antonio.bauza@uib.es, toni.frontera@uib.es;

Fax: +34 971 173426

Electronic Supplementary Information

CSD codes of Structures containing cyclopentasilanes

BUJJAT, BUJJIB, DUDSUS, ELAFIH, ELAFON, FOSDUO, GEDJIK, GEDJOQ, HINXEI, PEMPAA, QUZSAH.

CSD codes of Structures containing cyclohexasilanes

AHASEJ, AHASIN, AZAZUX, AZEBAJ, AZEBEN, AZEBIR, IKIGAL, KOTQER, KURMER, KURMIV, KURMOB, LECXAU, LECXIC, NENLOH, POWQOJ, POWQUP, POWRAW, POWREA, POWRIE, POWROK, QUZRUA, QUZSEL, VEYCOT.

Cartesian coordinates

5.

Cl	-2.5402889	1.8412998	2.2633480
Cl	-2.5404610	-1.8414984	2.2631026
Cl	-2.6637782	-1.9430962	-1.0632469
Cl	-2.6639590	1.9433086	-1.0629464
Cl	1.0222443	-3.1422897	-1.0488630
Cl	0.9676025	-2.9696156	2.2777308

Cl	1.0221213	3.1421254	-1.0490562
Cl	3.2855851	-0.0001395	-1.0728242
Cl	3.1473119	0.0000558	2.2528980
Cl	0.9676738	2.9697889	2.2775907
N	-0.0015539	0.0000124	-1.8041134
C	-0.0002796	0.0000139	-2.9461664
H	0.0016143	0.0000142	-4.0157419
Si	-1.6368009	1.1887647	0.5434904
Si	-1.6368003	-1.1887912	0.5434157
Si	0.6243746	-1.9221460	0.5501301
Si	2.0210377	0.0000193	0.5411449
Si	0.6243560	1.9221735	0.5501072

6.

Cl	-2.5564581	-1.8536539	-2.0037381
Cl	-2.5562292	1.8537181	-2.0037156
Cl	-2.6351941	1.9166809	1.3380616
Cl	-2.6348972	-1.9168053	1.3381240
Cl	1.0128167	3.1070969	1.3237380
Cl	0.9745405	2.9893318	-2.0175997
Cl	1.0129519	-3.1066563	1.3238246
Cl	3.2471395	-0.0004141	1.3465230
Cl	3.1657298	0.0000988	-1.9937839
Cl	0.9745563	-2.9895207	-2.0175929
Si	-1.6388096	-1.1903954	-0.2982490
Si	-1.6387618	1.1903980	-0.2981565
Si	0.6251971	1.9253801	-0.3040196

Si	2.0229979	0.0000079	-0.2960027
Si	0.6251436	-1.9253444	-0.3041763
F	-0.0010773	0.0000408	1.9712330
H	0.0003536	0.0000368	2.8955302

7.

Cl	-2.6872285	1.9523858	1.8511424
Cl	-2.6872285	-1.9523858	1.8511424
Cl	-2.5840918	-1.8774526	-1.4386018
Cl	-2.5840918	1.8774526	-1.4386018
Cl	0.9870352	-3.0377821	-1.4386018
Cl	1.0264299	-3.1590265	1.8511424
Cl	0.9870352	3.0377821	-1.4386018
Cl	3.1941131	0.0000000	-1.4386018
Cl	3.3215971	0.0000000	1.8511424
Cl	1.0264299	3.1590265	1.8511424
Si	-1.6042817	1.1655789	0.2429080
Si	-1.6042817	-1.1655789	0.2429080
Si	0.6127811	-1.8859462	0.2429080
Si	1.9830012	0.0000000	0.2429080
Si	0.6127811	1.8859462	0.2429080
O	0.0000000	0.0000000	-1.1578548
H	0.0000000	0.0000000	-2.1193882

8.

Cl	-2.5384289	1.8442765	1.8051105
Cl	-2.5384289	-1.8442765	1.8051105

Cl	-2.7532352	-2.0003425	-1.4573397
Cl	-2.7532352	2.0003425	-1.4573397
Cl	1.0516423	-3.2366221	-1.4573397
Cl	0.9695936	-2.9841021	1.8051105
Cl	1.0516423	3.2366221	-1.4573397
Cl	3.4031859	0.0000000	-1.4573397
Cl	3.1376707	0.0000000	1.8051105
Cl	0.9695936	2.9841021	1.8051105
Si	-1.6187197	1.1760687	0.0546264
Si	-1.6187197	-1.1760687	0.0546264
Si	0.6182959	-1.9029191	0.0546264
Si	2.0008476	0.0000000	0.0546264
Si	0.6182959	1.9029191	0.0546264
Cl	0.0000000	0.0000000	-2.0119856

9.

Cl	-2.6755818	1.9404616	2.3291188
Cl	-2.6755818	-1.9404616	2.3291188
Cl	-2.7843415	-2.0233742	-1.1344422
Cl	-2.7843415	2.0233742	-1.1344422
Cl	1.0713938	-3.2692907	-1.1303512
Cl	1.0243904	-3.1381597	2.3329976
Cl	1.0713938	3.2692907	-1.1303512
Cl	3.4273637	0.0000000	-1.1401198
Cl	3.3065588	-0.0000000	2.3227276
Cl	1.0243904	3.1381598	2.3329976
Ge	-1.7104416	1.2420787	0.5485022

Ge	-1.7104416	-1.2420788	0.5485022
Ge	0.6516279	-2.0086560	0.5514825
Ge	2.1071789	-0.0000000	0.5486161
Ge	0.6516279	2.0086560	0.5514825
N	-0.0019705	-0.0000000	-1.7905353
C	0.0012899	0.0000000	-2.9326833
H	0.0054850	0.0000000	-4.0026210

10.

Cl	-2.6963450	-1.9555912	-2.0656852
Cl	-2.6963450	1.9555912	-2.0656852
Cl	-2.7501556	1.9942459	1.4129718
Cl	-2.7501556	-1.9942459	1.4129718
Cl	1.0597104	3.2262898	1.4115415
Cl	1.0346131	3.1664025	-2.0657946
Cl	1.0597104	-3.2262898	1.4115415
Cl	3.3869806	-0.0000000	1.4129915
Cl	3.3276457	-0.0000000	-2.0642736
Cl	1.0346131	-3.1664025	-2.0657946
Ge	-1.7119280	-1.2435745	-0.3040092
Ge	-1.7119280	1.2435745	-0.3040092
Ge	0.6529821	2.0119669	-0.3050684
Ge	2.1095195	0.0000000	-0.3059533
Ge	0.6529821	-2.0119669	-0.3050684
F	-0.0016306	0.0000000	1.9321386
H	-0.0002688	0.0000000	2.8571852

11.

Cl	-2.8353208	2.0599811	1.9165100
Cl	-2.8353208	-2.0599811	1.9165100
Cl	-2.6993263	-1.9611754	-1.5053908
Cl	-2.6993263	1.9611754	-1.5053908
Cl	1.0310509	-3.1732484	-1.5053908
Cl	1.0829962	-3.3331195	1.9165100
Cl	1.0310509	3.1732484	-1.5053908
Cl	3.3365509	0.0000000	-1.5053908
Cl	3.5046492	0.0000000	1.9165100
Cl	1.0829962	3.3331195	1.9165100
Ge	-1.6827671	1.2226018	0.2522309
Ge	-1.6827671	-1.2226018	0.2522309
Ge	0.6427598	-1.9782113	0.2522309
Ge	2.0800145	0.0000000	0.2522309
Ge	0.6427598	1.9782113	0.2522309
O	0.0000000	0.0000000	-1.1777842
H	0.0000000	0.0000000	-2.1389665

12.

Cl	-2.6640235	1.9355264	1.8791194
Cl	-2.6640235	-1.9355264	1.8791194
Cl	-2.8816770	-2.0936609	-1.5196830
Cl	-2.8816770	2.0936609	-1.5196830
Cl	1.1007027	-3.3876144	-1.5196830
Cl	1.0175664	-3.1317474	1.8791194
Cl	1.1007027	3.3876144	-1.5196830

Cl	3.5619486	0.0000000	-1.5196830
Cl	3.2929141	0.0000000	1.8791194
Cl	1.0175664	3.1317474	1.8791194
Cl	0.0000000	0.0000000	-2.0666886
Ge	-1.6952531	-1.2316735	0.0539016
Ge	-1.6952531	1.2316735	0.0539016
Ge	0.6475291	1.9928895	0.0539016
Ge	0.6475291	-1.9928895	0.0539016
Ge	2.0954481	0.0000000	0.0539016

13.

Si	-2.0646264	1.1909992	0.4647553
Si	-2.0646267	-1.1909996	0.4647668
Si	-0.0000002	-2.3808119	0.4647034
Si	2.0646263	-1.1909992	0.4647502
Si	2.0646268	1.1909997	0.4647719
Si	0.0000002	2.3808119	0.4646982
Cl	-3.0977317	1.7853522	2.1317157
Cl	-3.0977237	-1.7853397	2.1317370
Cl	-3.1081522	-1.7914520	-1.1972683
Cl	-0.0000070	-3.5839154	-1.1975482
Cl	3.1081401	-1.7914371	-1.1972976
Cl	3.1081582	1.7914554	-1.1972583
Cl	0.0000070	3.5839086	-1.1975582
Cl	3.0977160	1.7853353	2.1317485
Cl	-0.0000069	3.5713105	2.1322278
Cl	3.0977394	-1.7853566	2.1317042

Cl	0.0000070	-3.5713017	2.1322393
Cl	-3.1081460	1.7914405	-1.1972875
N	-0.0000000	0.0000000	-1.6798324
C	-0.0000000	0.0000000	-2.8228660
H	-0.0000000	0.0000000	-3.8929015

13A

Si	-2.0580492	1.1883119	-0.0000000
Si	-2.0580492	-1.1883119	0.0000000
Si	-0.0000000	-2.3762003	0.0000000
Si	2.0580492	-1.1883119	-0.0000000
Si	2.0580492	1.1883119	-0.0000000
Si	-0.0000000	2.3762003	0.0000000
Cl	-3.1102685	1.7943754	1.6586848
Cl	-3.1102685	-1.7943754	1.6586848
Cl	-3.1102685	-1.7943754	-1.6586848
Cl	0.0000000	-3.5899703	-1.6585243
Cl	3.1102685	-1.7943754	-1.6586848
Cl	3.1102685	1.7943754	-1.6586848
Cl	0.0000000	3.5899703	-1.6585243
Cl	3.1102685	1.7943754	1.6586848
Cl	0.0000000	3.5899703	1.6585243
Cl	3.1102685	-1.7943754	1.6586848
Cl	0.0000000	-3.5899703	1.6585243
Cl	-3.1102685	1.7943754	-1.6586848
N	-0.0000000	-0.0000000	-2.1571763
C	0.0000000	0.0000000	-3.3001977

H	-0.0000000	-0.0000000	-4.3700361
N	0.0000000	0.0000000	2.1571763
C	-0.0000000	-0.0000000	3.3001977
H	0.0000000	-0.0000000	4.3700361

14.

Si	-2.0668287	1.1922022	0.2550704
Si	-2.0668283	-1.1922018	0.2550494
Si	0.0000001	-2.3831427	0.2576259
Si	2.0668286	-1.1922021	0.2550625
Si	2.0668284	1.1922018	0.2550574
Si	-0.0000001	2.3831426	0.2576180
Cl	-3.1067416	1.7894314	1.9146486
Cl	-3.1067659	-1.7894517	1.9146047
Cl	-3.0892982	-1.7819433	-1.4218419
Cl	0.0000056	-3.5672158	-1.4162241
Cl	3.0893080	-1.7819552	-1.4218189
Cl	3.0893076	1.7819487	-1.4218263
Cl	-0.0000056	3.5672051	-1.4162396
Cl	3.1067540	1.7894447	1.9146227
Cl	0.0000055	3.5765381	1.9209528
Cl	3.1067535	-1.7894384	1.9146307
Cl	-0.0000055	-3.5765242	1.9209707
Cl	-3.0893174	1.7819606	-1.4218034
F	-0.0000000	0.0000000	-1.7954220
H	-0.0000000	0.0000000	-2.7207375

14A

Si	2.0625420	-1.1900095	0.0000004
Si	2.0625420	1.1900095	-0.0000004
Si	0.0000000	2.3790610	0.0000002
Si	-2.0625420	1.1900095	0.0000002
Si	-2.0625420	-1.1900095	-0.0000003
Si	0.0000000	-2.3790610	0.0000000
Cl	3.1018010	-1.7876905	1.6657098
Cl	3.1018015	1.7876911	1.6657085
Cl	3.1018009	1.7876906	-1.6657099
Cl	-0.0000003	3.5770182	-1.6660242
Cl	-3.1018013	1.7876911	-1.6657088
Cl	-3.1018010	-1.7876907	-1.6657096
Cl	0.0000003	-3.5770181	-1.6660244
Cl	-3.1018014	-1.7876911	1.6657087
Cl	-0.0000003	-3.5770181	1.6660244
Cl	-3.1018012	1.7876906	1.6657095
Cl	0.0000003	3.5770179	1.6660247
Cl	3.1018014	-1.7876912	-1.6657086
F	0.0000000	0.0000000	-2.0618460
F	-0.0000000	0.0000000	2.0618460
H	0.0000000	0.0000000	-2.9870163
H	-0.0000000	0.0000000	2.9870163

15.

Si	-2.0260625	1.1697477	0.2075135
Si	-2.0260625	-1.1697477	0.2075135
Si	0.0000000	-2.3394954	0.2075135

Si	2.0260625	-1.1697477	0.2075135
Si	2.0260625	1.1697477	0.2075135
Si	0.0000000	2.3394954	0.2075135
Cl	-3.1970323	1.8458074	1.7951630
Cl	-3.1970323	-1.8458074	1.7951630
Cl	-3.0336401	-1.7514730	-1.5037084
Cl	0.0000000	-3.5029459	-1.5037084
Cl	3.0336401	-1.7514730	-1.5037084
Cl	3.0336401	1.7514730	-1.5037084
Cl	0.0000000	3.5029459	-1.5037084
Cl	3.1970323	1.8458074	1.7951630
Cl	0.0000000	3.6916149	1.7951630
Cl	3.1970323	-1.8458074	1.7951630
Cl	0.0000000	-3.6916149	1.7951630
Cl	-3.0336401	1.7514730	-1.5037084
H	0.0000000	0.0000000	-1.9783750
O	-0.0000000	0.0000000	-1.0154334

16.

Si	-2.0430575	1.1795598	0.0821928
Si	-2.0430575	-1.1795598	0.0821928
Si	-0.0000000	-2.3591195	0.0821928
Si	2.0430575	-1.1795598	0.0821928
Si	2.0430575	1.1795598	0.0821928
Si	0.0000000	2.3591195	0.0821928
Cl	-3.1167925	1.7994810	1.7539301
Cl	-3.1167925	-1.7994810	1.7539301

Cl	-3.1419178	-1.8139871	-1.5415754
Cl	0.0000000	-3.6279742	-1.5415754
Cl	3.1419178	-1.8139871	-1.5415754
Cl	3.1419178	1.8139871	-1.5415754
Cl	0.0000000	3.6279742	-1.5415754
Cl	3.1167925	1.7994810	1.7539301
Cl	-0.0000000	3.5989619	1.7539301
Cl	3.1167925	-1.7994810	1.7539301
Cl	-0.0000000	-3.5989619	1.7539301
Cl	-3.1419178	1.8139871	-1.5415754
Cl	0.0000000	0.0000000	-1.7672852

17.

Ge	2.1474163	-1.2384967	0.4663774
Ge	2.1474163	1.2384967	0.4663774
Ge	-0.0000000	2.4762644	0.4687790
Ge	-2.1474163	1.2384967	0.4663774
Ge	-2.1474163	-1.2384967	0.4663774
Ge	-0.0000000	-2.4762644	0.4687790
Cl	3.2495050	-1.8680656	2.1907298
Cl	3.2495050	1.8680656	2.1907298
Cl	3.2311987	1.8601096	-1.2766232
Cl	0.0000000	3.7272613	-1.2725972
Cl	-3.2311987	1.8601096	-1.2766231
Cl	-3.2311987	-1.8601096	-1.2766232
Cl	-0.0000000	-3.7272613	-1.2725972
Cl	-3.2495050	-1.8680656	2.1907298

Cl	-0.0000000	-3.7397626	2.1969679
Cl	-3.2495050	1.8680656	2.1907299
Cl	-0.0000000	3.7397626	2.1969679
Cl	3.2311987	-1.8601096	-1.2766232
N	-0.0000000	0.0000000	-1.6504263
C	0.0000000	-0.0000000	-2.7936451
H	0.0000001	-0.0000001	-3.8641626

18.

Cl	-3.2585669	1.8722262	1.9745486
Cl	-3.2585669	-1.8722262	1.9745486
Cl	-3.2113861	-1.8502734	-1.5000405
Cl	0.0000001	-3.7097759	-1.4898685
Cl	3.2113860	-1.8502734	-1.5000405
Cl	3.2113860	1.8502734	-1.5000405
Cl	0.0000001	3.7097759	-1.4898685
Cl	3.2585668	1.8722262	1.9745486
Cl	0.0000001	3.7443350	1.9866169
Cl	3.2585668	-1.8722262	1.9745486
Cl	0.0000000	-3.7443350	1.9866169
Cl	-3.2113861	1.8502734	-1.5000405
F	-0.0000000	-0.0000000	-1.7614966
H	-0.0000000	-0.0000000	-2.6875643
Ge	-2.1494360	-1.2394718	0.2580279
Ge	-2.1494360	1.2394718	0.2580279
Ge	-0.0000000	2.4776936	0.2627086
Ge	2.1494360	1.2394718	0.2580279

Ge	2.1494360	-1.2394718	0.2580279
Ge	-0.0000000	-2.4776936	0.2627086

19.

Ge	-2.1137407	1.2203688	0.2139717
Ge	-2.1137407	-1.2203688	0.2139717
Ge	0.0000000	-2.4407375	0.2139717
Ge	2.1137407	-1.2203688	0.2139717
Ge	2.1137407	1.2203688	0.2139717
Ge	0.0000000	2.4407375	0.2139717
Cl	-3.3590389	1.9393420	1.8533455
Cl	-3.3590389	-1.9393420	1.8533455
Cl	-3.1546562	-1.8213416	-1.5796288
Cl	0.0000000	-3.6426832	-1.5796288
Cl	3.1546562	-1.8213416	-1.5796288
Cl	3.1546562	1.8213416	-1.5796288
Cl	0.0000000	3.6426832	-1.5796288
Cl	3.3590389	1.9393420	1.8533455
Cl	0.0000000	3.8786840	1.8533455
Cl	3.3590389	-1.9393420	1.8533455
Cl	0.0000000	-3.8786840	1.8533455
Cl	-3.1546562	1.8213416	-1.5796288
H	-0.0000000	0.0000000	-1.9445440
O	0.0000000	0.0000000	-0.9815863

20.

Ge	-2.1304150	1.2299957	0.0801984

Ge	-2.1304150	-1.2299957	0.0801984
Ge	0.0000000	-2.4599913	0.0801984
Ge	2.1304150	-1.2299957	0.0801984
Ge	2.1304150	1.2299957	0.0801984
Ge	0.0000000	2.4599913	0.0801984
Cl	-3.2540599	1.8787323	1.8266406
Cl	-3.2540599	-1.8787323	1.8266406
Cl	-3.2832362	-1.8955773	-1.6085968
Cl	0.0000000	-3.7911546	-1.6085968
Cl	3.2832362	-1.8955773	-1.6085968
Cl	3.2832362	1.8955773	-1.6085968
Cl	-0.0000000	3.7911546	-1.6085968
Cl	3.2540599	1.8787323	1.8266406
Cl	0.0000000	3.7574647	1.8266406
Cl	3.2540599	-1.8787323	1.8266406
Cl	0.0000000	-3.7574647	1.8266406
Cl	-3.2832362	1.8955773	-1.6085968
Cl	-0.0000000	0.0000000	-1.7894534
